# Synthetic Mimics for Nitrogen-Based Polycyclic Aromatic Hydrocarbon Cosmic Dust: Preparation and Preliminary Impact Ionization Mass Spectrometry Studies

**DOI:** 10.1021/jacs.5c18079

**Published:** 2026-03-23

**Authors:** Min Zeng, Derek H. H. Chan, Steven P. Armes, Rebecca Mikula, John Fontanese, Zoltan Sternovsky

**Affiliations:** † School of Mathematical and Physical Sciences, 7315 University of Sheffield, Brook Hill, Sheffield, South Yorkshire S3 7HF, U.K.; ‡ Laboratory for Atmospheric and Space Physics, 1877 University of Colorado, Boulder, Colorado 80303, United States; § Smead Aerospace Engineering Sciences Department, University of Colorado, Boulder, Colorado 80303, United States

## Abstract

We report the preparation of the first synthetic mimic for submicron-sized nitrogen-based polycyclic aromatic hydrocarbon (PANH) cosmic dust. Melting point phase diagrams were constructed for two binary mixtures comprising *N*-phenylcarbazole (mp 96 °C) with either *N*-ethylcarbazole (mp 71 °C) or *N*-propylcarbazole (mp 50 °C) to identify their respective eutectic compositions. Each eutectic composition was then processed above its eutectic temperature via hot emulsification using a commercial water-soluble polymeric emulsifier: high-shear homogenization produced polydisperse molten PANH droplets of approximately 60–70 μm diameter. Each precursor emulsion was then subjected to high-pressure microfluidization to produce much finer submicron-sized PANH droplets. On cooling to 20 °C, these hybrid PANH microparticles were coated with an ultrathin overlayer of polypyrrole (PPy). This electrically conductive coating enabled the efficient accumulation of surface charge, which in turn allowed the electrostatic acceleration of such PPy-coated PANH microparticles up to the hypervelocity regime using a high-voltage dust accelerator. Firing such PPy-coated PANH microparticles into a gold target at 1.9–5.0 km s^–1^ led to their impact ionization and the in situ generation of an ionic plasma. Subsequent impact ionization mass spectrometry analysis confirmed the formation of the characteristic parent cations for *N*-phenylcarbazole and either *N*-propylcarbazole or *N*-ethylcarbazole, respectively. Such laboratory-based experiments augur well for the unambiguous identification of PANH-based cosmic dust by next-generation impact ionization mass spectrometers to be deployed in current and future space missions.

## Graphical abstract

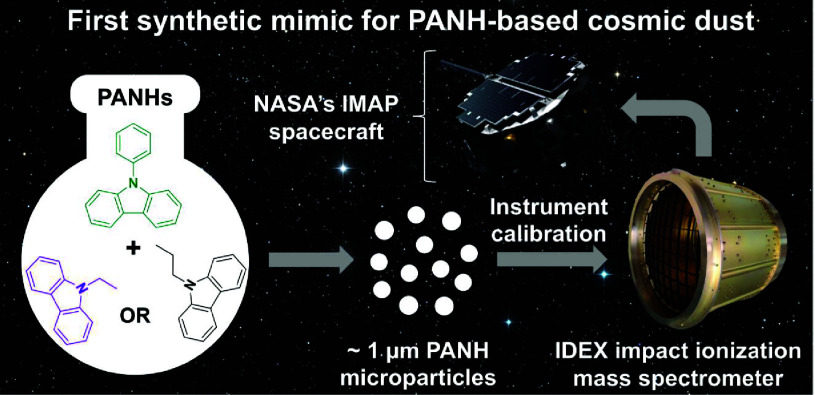

## Introduction

Polycyclic aromatic hydrocarbons (PAHs) have been detected in meteorites,^1^ comets,^2^ on Titan,^3^ and within interplanetary cosmic dust grains.^4^ They are an important source of carbon^5,6^ in the known universe, which has potential implications for the origin of life.^7^ It has also been suggested that PAHs influence the evolution of galaxies and the formation of planets and stars.^5,8–11^ PAHs were first identified within the interstellar medium by Tielens et al., who observed a strong infrared emission band at around 6.2 μm.^8^ Subsequently, this spectral feature has been attributed to the presence of nitrogen-containing PAH molecules (sometimes denoted as PANH).^12–19^ Recently, plausible progenitors for PANH molecules (e.g., benzonitrile^20^ and cyanopyrene^21,22^ ) have been detected within the interstellar medium. Together, these observations suggest that PANHs are distributed throughout the Universe. If so, it would be both useful and timely to design synthetic mimics for PANH cosmic dust for the calibration of the next generation of impact ionization mass spectrometers (a.k.a. cosmic dust analyzers) to be deployed in future space missions.^23–26^

Accordingly, we sought to prepare the first synthetic mimics for PANH cosmic dust to supplement the various PAH-based mimics that have been recently reported.^27–29^ Taking into account the toxicity, cost and appropriate physical properties (e.g., relatively low melting point and minimal aqueous solubility) of a library of putative molecules, we chose to focus on *N*-ethylcarbazole, *N*-propylcarbazole and *N*-phenylcarbazole (see Scheme 1). In 2024 we reported the preparation of phenanthrene/pyrene hybrid microparticles via hot emulsification processing.^29^ A key aspect of this prior study was the construction of a melting point phase diagram to identify the eutectic composition (75 mol % phenanthrene), which ensured minimal change in chemical composition during subsequent processing.^29^ However, only relatively large microparticles of 11 to 279 μm diameter were prepared by this approach. These synthetic mimics are useful for impact crater studies using a light gas gun,^30,31^ but unfortunately the hypervelocity regime is not accessible for such massive microparticles so their impact ionization mass spectra could not be recorded.^29^

**Scheme 1:**
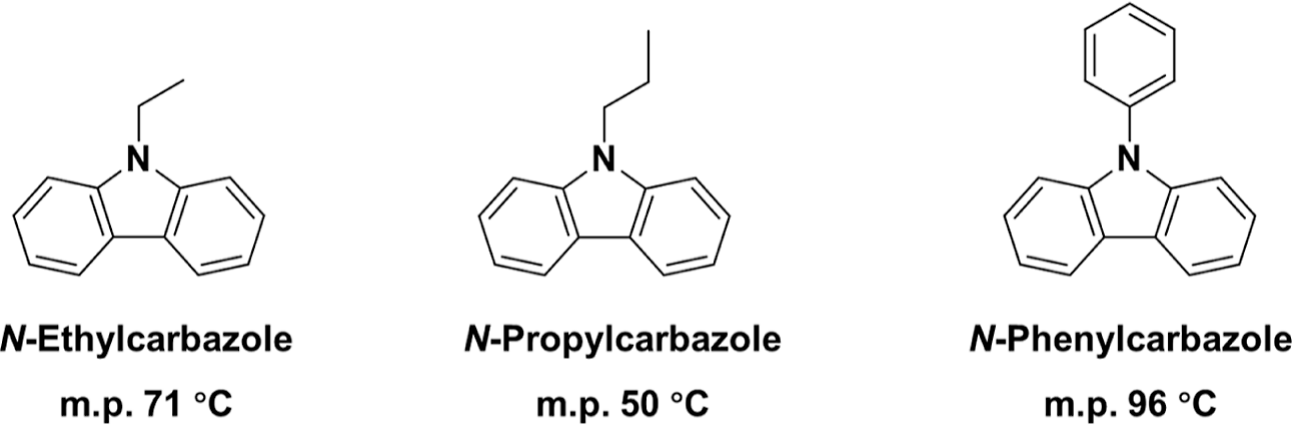
Chemical Structures of *N*-Ethylcarbazole, *N*-Propylcarbazole and *N*-Phenylcarbazole and Their Corresponding Melting Points

Recently, we reported using a similar hot emulsification route to prepare relatively large PANH-based microparticles of 12 to 273 μm diameter based on benzo­[*h*]­quinoline.^32^ Unfortunately, it is not feasible to prepare the corresponding submicron-sized benzo­[*h*]­quinoline microparticles required for impact ionization mass spectrometry studies because the relatively high solubility of benzo­[*h*]­quinoline in acidic media would cause microparticle dissolution prior to the polypyrrole coating step. Instead, we have extended this approach by (i) using either *N*-ethylcarbazole/*N*-phenylcarbazole or *N*-propylcarbazole/*N*-phenylcarbazole binary mixtures at their respective eutectic compositions to produce hybrid PANH molten droplets and (ii) employing high-pressure microfluidization to convert these initial coarse emulsions into much finer molten droplets of less than 1 μm diameter (see Figure 1) that form hybrid PANH microparticles on cooling.

**Figure 1:**
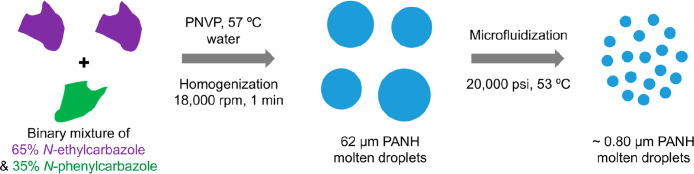
Schematic representation of the hot emulsification of a binary mixture comprising 65 mol % *N*-ethylcarbazole and 35 mol % *N*-phenylcarbazole using poly­(*N*-vinylpyrrolidone) [PNVP] as a water-soluble polymeric emulsifier at 57 °C to produce an initial coarse oil-in-water emulsion (step 1). Subsequent high-pressure microfluidization of this precursor emulsion at 53 °C produces a much finer emulsion comprising hybrid PANH droplets of ca. 0.80 μm diameter (step 2). Essentially the same protocol was used to prepare *N*-propylcarbazole/*N*-phenylcarbazole microparticles comprising 71 mol % *N*-propylcarbazole (see Experimental section in the Supporting Information for further details).

In principle, such microparticles can be coated with an ultrathin overlayer of an electrically conductive polymer (polypyrrole)^33^ to produce the first synthetic mimic for PANH-based cosmic dust that can be accelerated up to the hypervelocity regime (>1 km s^–1^) using an electrostatic dust accelerator.^30,34,35^ Such speeds correspond to those typically encountered for cosmic dust. If such fast-moving organic dust particles strike a suitable metal target, their kinetic energy is sufficiently high to cause instantaneous molecular fragmentation and the generation of an ionic plasma.^35^ Hence their chemical composition can be analyzed in situ via impact ionization mass spectrometry.^35^ This detection principle was the basis for the Cosmic Dust Analyzer (CDA) on the now-defunct CASSINI spacecraft.^36^ However, this instrument required careful calibration with a series of model micron-sized projectiles of known chemical composition prior to the interpretation of data obtained for cosmic dust particles originating from the Saturnian ring system.^36,37^ Thus, the aim of the current study was to produce the first suitable synthetic mimic for PANH-based cosmic dust to enable calibration of state-of-the-art impact ionization mass spectrometers such as the SUrface Dust Analyzer (SUDA)^25^ and Interstellar Dust Experiment (IDEX)^26^ that form part of the current Europa Clipper^38^ and Interstellar Mapping and Acceleration Probe (IMAP)^23,26^ space missions, respectively. Both SUDA and IDEX are reflectron-type instruments with upper limit measurable masses of more than 200 Da. The main objective for SUDA is the analysis of dust ejecta from Europa. This Jovian moon is believed to harbor subsurface liquid water between its ice crust and silicate core so its potential inhabitability for life is of considerable interest. In contrast, IDEX has been designed to determine the flux, speed, mass and chemical composition of both interstellar and interplanetary dust while IMAP is stationed between the Earth and the Sun. IDEX has been optimized to detect dust grain masses ranging from 0.2 to 50 pg.

## Results and Discussion

### Background and Context

In 2005, the Cassini began a series of flybys to sample the icy plumes emanating from the subsurface ocean of the Saturnian moon, Encedalus.^39–42^ Its CDA instrument recorded many impact ionization mass spectra that are still being analyzed two decades later. Important findings include the identification of organic macromolecular species^43^ and phosphates.^44^ Very recently, various small organic molecules have also been reported, including nitrogen-based heterocycles such as substituted pyridines.^37^ Moreover, carbazole (mp ∼ 245 °C) has just been detected within dust samples retrieved from the asteroid Bennu.^45^ Herein we have taken a pragmatic approach and focused on N-substituted carbazole derivatives whose physical properties allow established processing techniques to be applied for the facile generation of spherical microparticles. More specifically, hot emulsification is combined with high-shear homogenization to produce an initial coarse emulsion comprising polydisperse PANH droplets. Recently, the same approach has been utilized to prepare relatively large PAH microparticles comprising either phenanthrene^28^ or 75:25 phenanthrene/pyrene.^29^

One important novel aspect of the current study is the use of high-pressure microfluidization as a second processing step. This technique has been widely used to prepare various types of nanoemulsions,^46–49^ but has never previously been applied for the preparation of synthetic mimics of cosmic dust. In principle, the judicious use of the eutectic composition for pairs of the three substituted carbazoles shown in Scheme 1 should ensure that there is no compositional variation during such processing. Thus, each individual microparticle should have precisely the same (eutectic) chemical composition. This concept has already been validated for 75:25 phenanthrene/pyrene hybrid microparticles.^29^ This aspect is critical for the desired impact ionization mass spectrometry calibration studies, for which each fast-moving microparticle constitutes a separate experiment.^35,50^

### Melting Point Phase Diagram for a Series of *N*-Ethylcarbazole/*N*-Phenylcarbazole Binary Mixtures

Figure 2 shows a melting point phase diagram constructed for a series of *N*-ethylcarbazole/*N*-phenylcarbazole binary mixtures. Notably, the eutectic temperature of a 65:35 *N*-ethylcarbazole/*N*-phenylcarbazole mixture is approximately 47 °C, which is significantly lower than the melting point of either *N*-ethylcarbazole (71 °C) or *N*-phenylcarbazole (96 °C) alone. This melting point depression enables emulsification of this eutectic composition via high-shear homogenization in aqueous solution under relatively mild conditions (see Figure 1). We envisaged that the relatively low eutectic temperature should allow further processing via high-pressure microfluidization. Interestingly, cooling at the eutectic composition led to no recrystallization on the time scale of the DSC experiment (see Figure S1). Stark et al. reported similar observations for binary mixtures of *N*-ethylcarbazole with other *N*-alkylcarbazoles and attributed this phenomenon to very slow recrystallization kinetics.^51^

**Figure 2:**
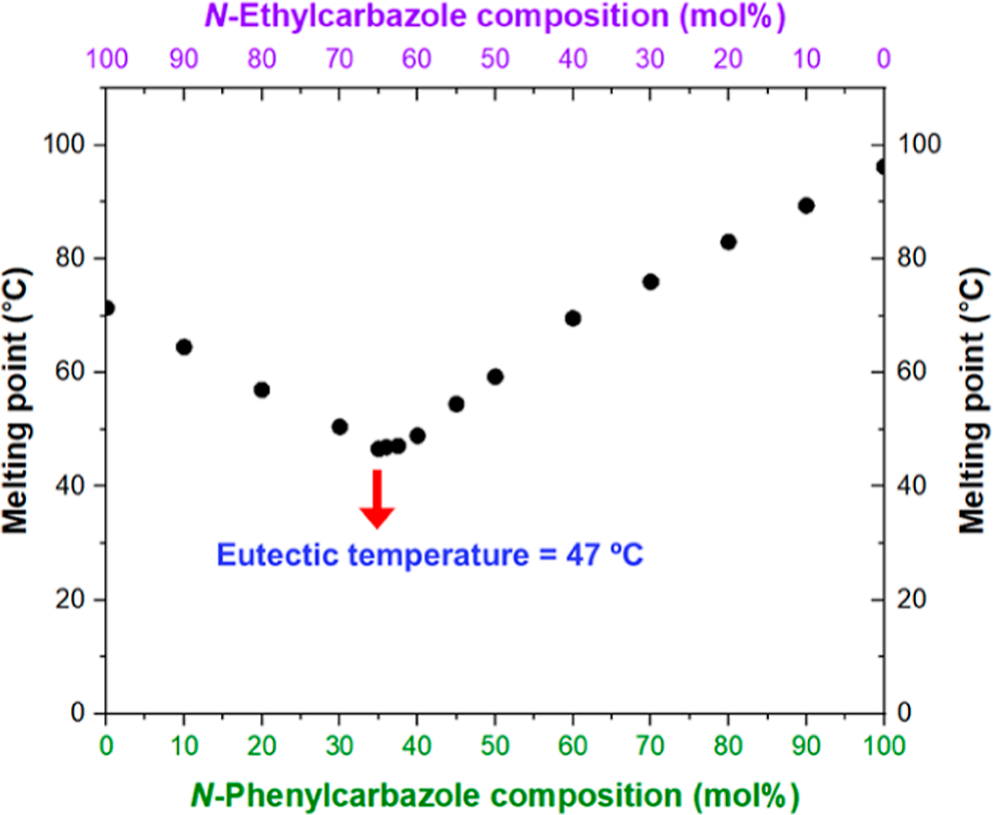
Melting point phase diagram constructed for a series of binary mixtures of *N*-ethylcarbazole and *N*-phenylcarbazole. The eutectic composition corresponds to 65 mol % *N*-ethylcarbazole, which has a eutectic temperature of 47 °C.

The synthetic route used to produce hybrid PANH microparticles of less than 1 μm diameter is shown in Figure 1. A molten binary mixture of 65:35 *N*-ethylcarbazole/*N*-phenylcarbazole was heated in water at 57 °C in the presence of a commercial water-soluble polymeric emulsifier (PNVP). High-shear homogenization of this hot mixture afforded a relatively coarse oil-in-water emulsion (Figure 3a).

**Figure 3:**
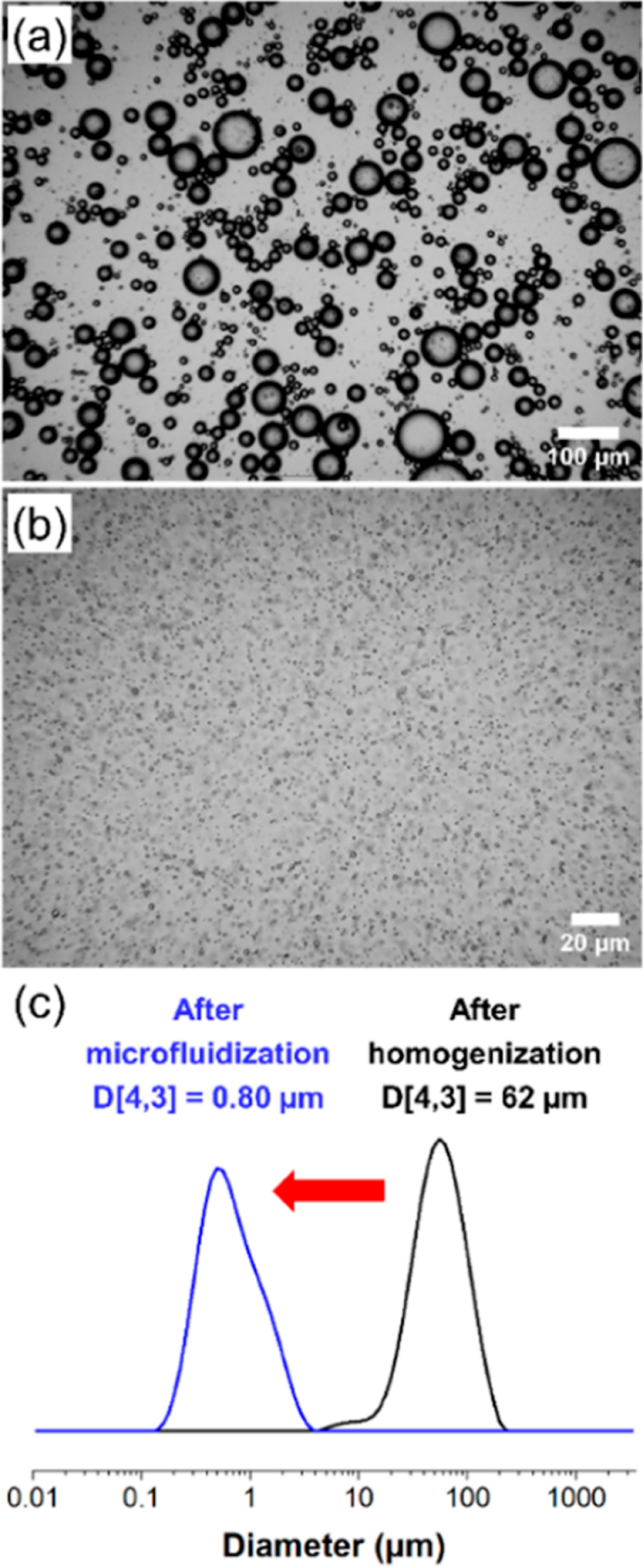
(a) Representative optical microscopy image recorded for the initial coarse PANH emulsion droplets (comprising 65 mol % *N*-ethylcarbazole and 35 mol % *N*-phenylcarbazole) obtained after homogenization (18,000 rpm for 1 min at 57 °C). (b) Representative optical microscopy image recorded for the corresponding much finer PANH emulsion droplets obtained after high-pressure microfluidization (one pass at 20,000 psi). (b) Laser diffraction particle size distribution recorded for the initial coarse PANH droplets (black curve) and the much finer PANH droplets obtained after high-pressure microfluidization using an LV1 microfluidizer (blue curve).

Laser diffraction analysis indicated a volume-average diameter of 62 μm for these initial PANH droplets (Figure 3c). This precursor emulsion was then subjected to high-pressure microfluidization at 53 °C to produce much finer droplets. After a single pass through an LV1 microfluidizer at an applied pressure of 20,000 psi (Figure 3b), laser diffraction analysis indicated a volume-average diameter of 0.80 μm (Figure 3c). These 0.80 μm PANH microparticles were then coated with an electrically conductive polymer, polypyrrole (PPy), using a well-known aqueous deposition protocol at 20 °C (Figure 4).^33,52^ Rather than FeCl_3_,^53^ ammonium persulfate [(NH_4_)_2_S_2_O_8_] was selected as the oxidant to minimize the reaction time.^54^ A (NH_4_)_2_S_2_O_8_/pyrrole molar ratio of 0.58 was chosen: this corresponds to a two-fold excess of pyrrole, which ensures that the oxidant is fully consumed.^28^ Such conditions should minimize the possibility of surface oxidation of the PANH microparticles. The oxidative polymerization of pyrrole was conducted in aqueous media at 20 °C for 10 min. A PPy mass loading of 7.4% was targeted, which corresponds to a mean PPy overlayer thickness of 8 nm.^33^ The resulting PPy-coated PANH microparticles were isolated by centrifugation and dried in a vacuum oven at 20 °C overnight to afford the final microparticles as a fine black powder. To confirm the anticipated core–shell morphology of the PPy-coated PANH microparticles, the PANH component was removed by solvent extraction using acetone (Figure 4). A mass balance experiment indicated a PPy mass loading of 10.7%, which corresponds to a PPy overlayer thickness of 11.4 nm. The discrepancy between the target and actual mass loading is ascribed to partial loss of material during the high-pressure microfluidization processing step. Nevertheless, the PPy overlayer remains a relatively minor component of the PANH microparticles, which is important for the interpretation of their impact ionization mass spectra (see later).

**Figure 4:**
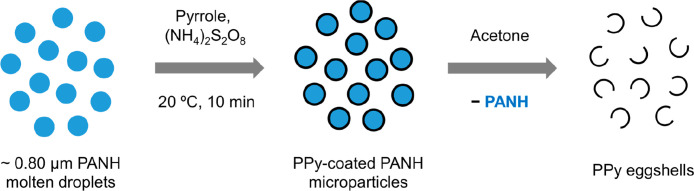
Schematic representation of the deposition of an ultrathin overlayer of polypyrrole onto 65:35 *N*-ethylcarbazole/*N*-phenylcarbazole molten droplets of ca. 0.80 μm diameter via oxidative polymerization of pyrrole in aqueous solution at 20 °C (step 1). Subsequent acetone extraction of the underlying cores from polypyrrole-coated 65:35 *N*-ethylcarbazole/*N*-phenylcarbazole microparticles of ca. 0.80 μm diameter to afford residual polypyrrole eggshells (step 2). This solvent extraction experiment provides strong evidence for the core–shell nature of the original polypyrrole-coated hybrid PANH microparticles (see Figures 5 and 6c).

Assigned FT-IR spectra recorded for the PPy-coated 65:35 *N*-ethylcarbazole/*N*-phenylcarbazole microparticles before and after acetone extraction of their PANH component are shown in Figure 5, along with reference spectra recorded for a binary mixture of 65:35 *N*-ethylcarbazole/*N*-phenylcarbazole crystals and PPy bulk powder. The strong broad bands at 1546 cm^–1^ (C=C stretch), 1042 cm^–1^ (C–H in-plane bend) and 898 cm^–1^ (C–H out-of-plane bend) observed for the PPy-coated PANH microparticles (see blue shaded regions in spectrum b) are also observed in the PPy bulk powder spectrum^55–57^ and hence provide evidence for the polypyrrole overlayer. Notably, the lack of any spectral features attributable to either *N*-ethylcarbazole or *N*-phenylcarbazole at 2900–3100, 1596, 1230, or 720 cm^–1^ confirms that the acetone extraction protocol completely removed these two components, leaving only the acetone-insoluble polypyrrole residues (see Figure 5).

**Figure 5:**
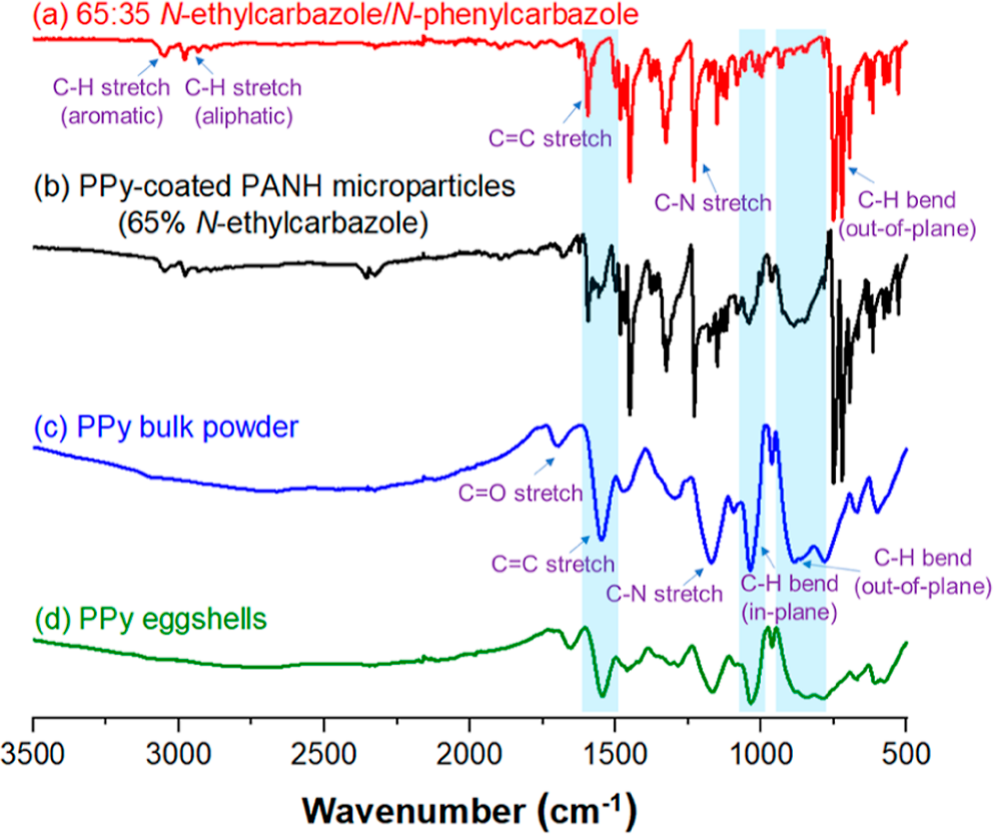
FT-IR spectra recorded in transmission mode for: (a) a binary mixture of *N*-ethylcarbazole/*N*-phenylcarbazole crystals comprising 65 mol % *N*-ethylcarbazole; (b) polypyrrole-coated 65:35 *N*-ethylcarbazole/*N*-phenylcarbazole hybrid microparticles of ca. 0.80 μm diameter; (c) polypyrrole bulk powder prepared using the (NH_4_)_2_S_2_O_8_ oxidant; (d) residual polypyrrole eggshells obtained after selective dissolution of the underlying hybrid PANH cores of such microparticles using acetone.

Figure 6a shows an optical microscopy image recorded for the PPy-coated PANH microparticles after their redispersal in water, while Figure 6b shows an SEM image recorded for the dried PPy-coated PANH microparticles. The latter image reveals the presence of microparticle aggregates, as well as individual microparticles. This is not unexpected given that PPy has a relatively high Hamaker constant.^58,59^ Figure 6c shows an SEM image recorded for the remaining acetone-insoluble PPy residues. The observed eggshell-like structure provides strong evidence for the core–shell morphology of the original PPy-coated PANH microparticles. Similar findings have been reported for PPy-coated polystyrene latex particles of comparable size.^60^

**Figure 6:**
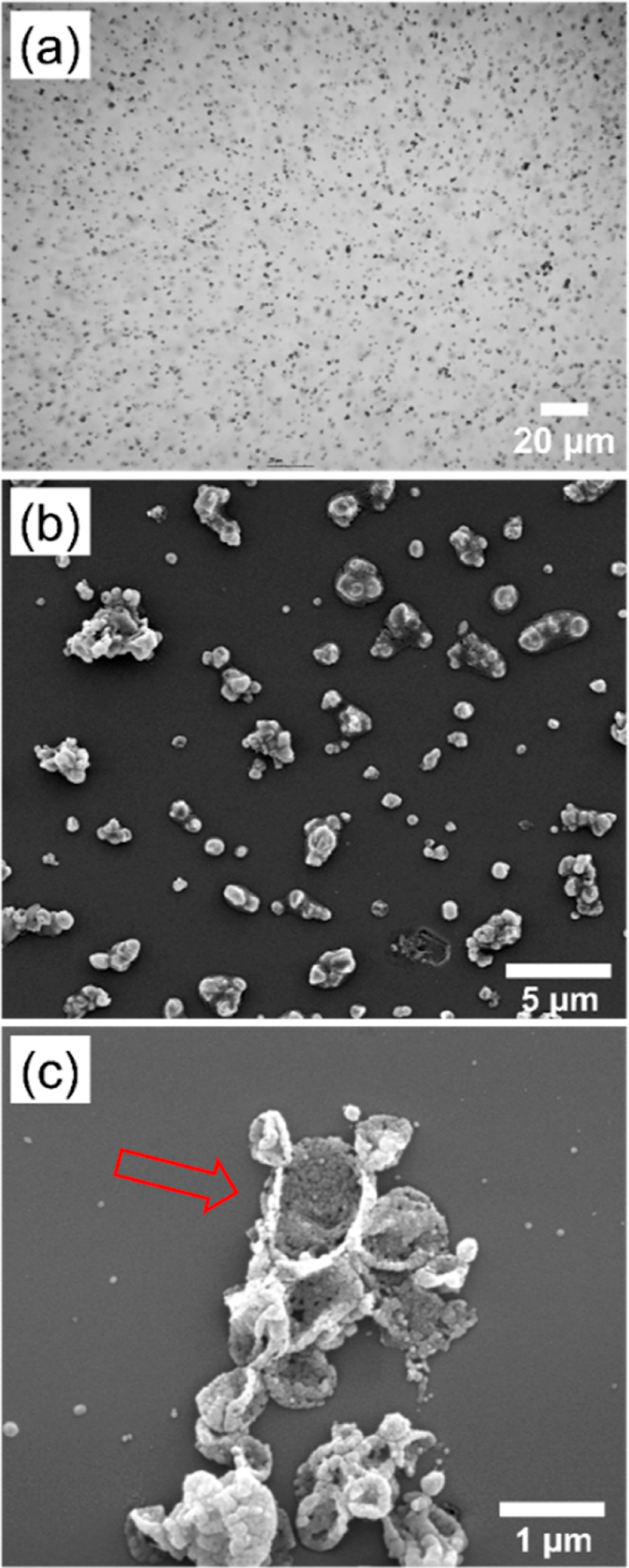
(a) Optical microscopy image obtained for polypyrrole-coated hybrid *N*-ethylcarbazole/*N*-phenylcarbazole microparticles comprising 65 mol % *N*-ethylcarbazole. Scanning electron microscopy images recorded for (b) polypyrrole-coated hybrid *N*-ethylcarbazole/*N*-phenylcarbazole microparticles and (c) residual polypyrrole eggshells obtained after selective dissolution of the underlying hybrid *N*-ethylcarbazole/*N*-phenylcarbazole cores using acetone. The red arrow indicates a relatively large polypyrrole eggshell.

A DSC curve recorded for the PPy-coated 65:35 *N*-ethylcarbazole/*N*-phenylcarbazole microparticles is shown in Figure S2. During the initial heating run (black curve), a single sharp endotherm is observed at 40 °C. During the cooling run (blue curve), there is little or no evidence for recrystallization even after cooling to −35 °C. During the second heating run (red curve), the broad exotherm between 0 and 30 °C is attributed to the relatively rough inner surface of the polypyrrole overlayer (see Figure 6c), which leads to partial (∼50%) crystallization of the hybrid PANH cores via heterogeneous nucleation.^61^ Subsequently, a sharp endotherm is observed at 40 °C, along with a weak shoulder at around 44 °C. This latter feature is attributed to slightly delayed melting for the crystalline domain that is in close proximity with the PPy shell (see Figure S2).

A second melting point phase diagram was constructed for a series of *N*-propylcarbazole/*N*-phenylcarbazole binary mixtures (Figure 7a). The eutectic composition of 71 mol % *N*-propylcarbazole has a eutectic temperature of 37 °C, which is significantly lower than the melting point of either *N*-propylcarbazole (50 °C) or *N*-phenylcarbazole (96 °C) alone. Again, this pronounced melting point depression enables emulsification of this eutectic composition via high-shear homogenization to produce hybrid PANH droplets with a volume-average diameter of 67 μm (Figures S3 and S4). Subsequent high-pressure microfluidization of this coarse precursor emulsion under relatively mild conditions produces much finer droplets with a volume-average diameter of 0.78 μm, which are then coated with PPy (Figures S3 and S4).

**Figure 7:**
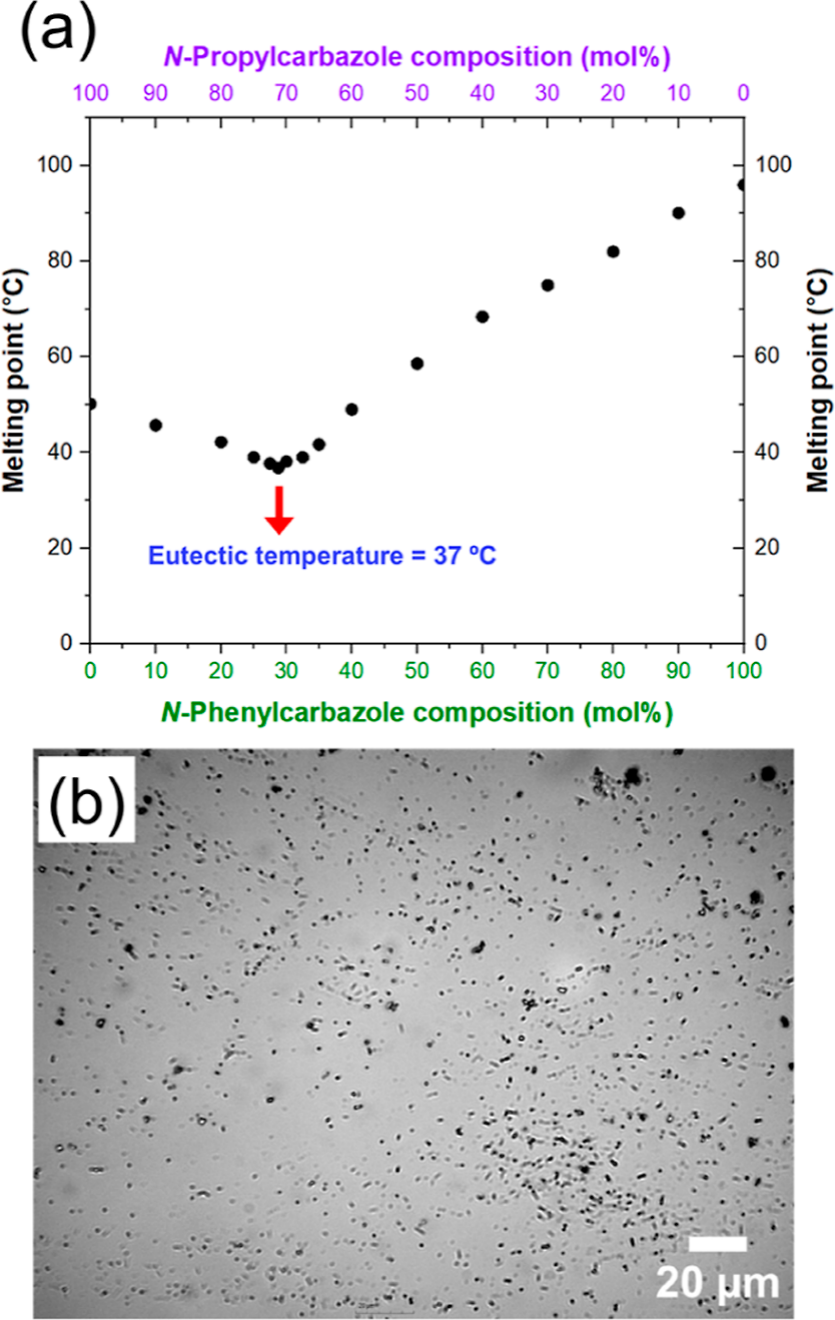
(a) Melting point phase diagram constructed for a series of binary mixtures of *N*-propylcarbazole and *N*-phenylcarbazole. The eutectic composition corresponds to 71 mol % *N*-propylcarbazole, which has a eutectic melting point of 37 °C. (b) Optical microscopy image obtained for polypyrrole-coated hybrid *N*-propylcarbazole/*N*-phenylcarbazole microparticles comprising 71 mol % *N*-propylcarbazole.

A representative optical micrograph obtained for the final PPy-coated 71:29 *N*-ethylcarbazole/*N*-phenylcarbazole microparticles is shown in Figure 7b. FT-IR spectra recorded for these microparticles before and after acetone extraction of their PANH cores are shown in Figure S5, along with reference spectra for a binary mixture of 71:29 *N*-ethylcarbazole/*N*-phenylcarbazole crystals and PPy bulk powder. The strong broad bands observed at 1546, 1042, and 898 cm^–1^ for the PPy-coated PANH microparticles are also present in the PPy bulk powder spectrum and hence provide good evidence for the PPy overlayer. This electrically conductive coating allowed the efficient accumulation of surface charge, which in turn enabled the electrostatic acceleration of such PPy-coated PANH microparticles up to the hypervelocity regime (>1 km s^–1^) that typifies the behavior of cosmic dust using a high-voltage dust accelerator (see Figure 8a).

**Figure 8:**
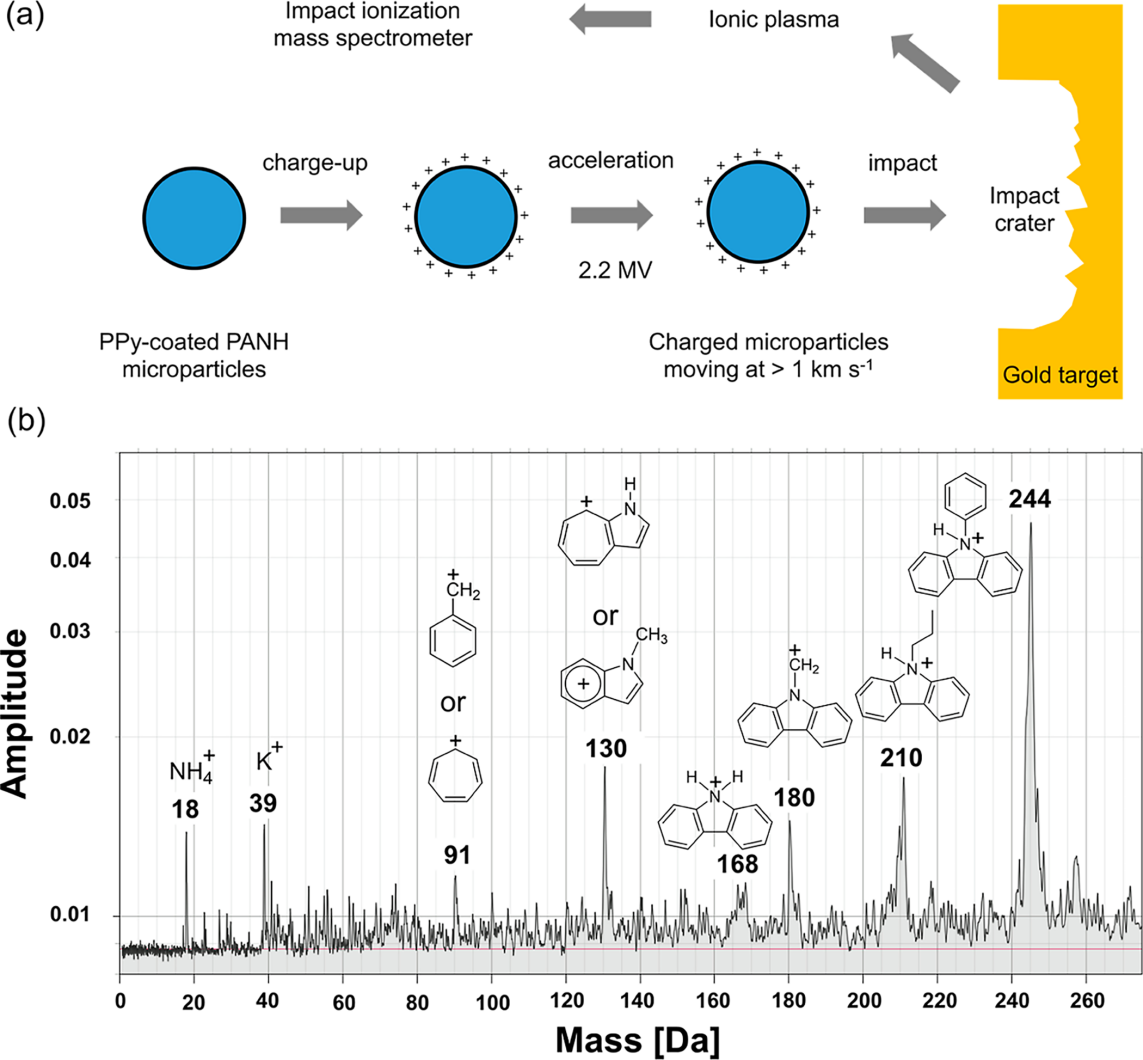
(a) Schematic representation of the electrostatic acceleration of polypyrrole-coated 71:29 *N*-propylcarbazole/*N*-phenylcarbazole PANH microparticles using a high-voltage dust accelerator [N.B. Whether the depicted impact crater is actually formed will depend on both the impact speed and the microparticle mass]. (b) Impact ionization mass spectrum recorded after firing a polypyrrole-coated 71:29 *N*-propylcarbazole/*N*-phenylcarbazole microparticle (mass = 2.5 picograms; mean diameter = 1.6 μm) into a gold target at 3.6 km s^–1^ using a 3 MV pelletron dust accelerator.

Preliminary impact ionization experiments were conducted using a 3 MV pelletron dust accelerator at the University of Colorado.^62^ Polypyrrole-coated 71:29 *N*-propylcarbazole/*N*-phenylcarbazole microparticles with a volume-average diameter of 0.78 μm were accelerated and fired at a gold target. Bearing in mind our recent study of the impact ionization behavior of anthracene microparticles,^27,35^ experiments performed at a sufficiently low hypervelocity were expected to generate the two parent cations associated with *N*-propylcarbazole and *N*-phenylcarbazole respectively, with minimal molecular fragmentation of either species. In this ideal scenario, the relative intensity of the two species should be 71:29. In contrast, an individual microparticle with a mass of approximately 2.5 picograms (corresponding to a mean diameter of around 1.6 μm) impacting at 3.6 km s^–1^ produced the impact ionization mass spectrum shown in Figure 8b. At this relatively low hypervelocity, there is insufficient kinetic energy to induce extensive molecular fragmentation so only (some of) the weaker chemical bonds are cleaved. Thus the pendant aliphatic group in *N*-propylcarbazole is susceptible to bond cleavage, whereas *N*-phenylcarbazole exhibits significantly greater chemical stability. The latter component is detected as a protonated parent cation at 244 Da with no obvious associated molecular fragments. In contrast, several lower mass signals corresponding to the (partial) fragmentation of *N*-propylcarbazole are detected at 180, 168, 130, and 91 Da, in addition to the protonated parent cation at 210 Da [N.B. The mass accuracy is estimated to be approximately ±1 Da for the higher masses]. The 180 and 168 Da signals are assigned to cleavage of an ethyl or propyl group, respectively. The 130 Da signal is tentatively assigned to a cationic methylated indole species, while the relatively minor signal at 91 Da is most likely due to the formation of tropylium cation.^37,50^ A second impact ionization mass spectrum recorded for an microparticle impinging at 1.9 km s^–1^ is provided in Figure S6. This spectrum is comparable to that shown in Figure 8b. However, additional mass signals are observed at 258 and 224 Da. These features are attributed to methylated derivatives of the two parent cations (see assigned molecular structures in Figure S6), while associated methylated molecular fragments are also observed at 144 and 88 Da. This suggests that plasma chemistry—in this case methylation of the nitrogen atom within each N-substituted carbazole—can affect the observed mass spectra under certain circumstances. In principle, this observation complicates the unambiguous analysis of PANH-based cosmic dust because it makes the identification of parent cations more problematic. At first sight, it may seem odd that such plasma chemistry is observed for an impact at 1.9 km s^–1^ yet not for the microparticle impinging at 3.6 km s^–1^. However, the mass of the former microparticle (23 pg) is almost an order of magnitude higher than that of the latter (2.5 pg). Thus, the slower microparticle actually has a kinetic energy approximately 2.6 times greater than that of the faster microparticle, which may explain why partial methylation of the two parent cations (and their associated fragments) only occurs in the former case.

Finally, a polypyrrole-coated 65:35 *N*-ethylcarbazole/*N*-phenylcarbazole microparticle with a diameter of 0.95 μm (mass = 0.55 pg) was fired into a gold target at 5.0 km s^–1^ (see Figure S7). As expected, the protonated parent cations for *N*-ethylcarbazole and *N*-phenylcarbazole are detected at 196 and ∼243–244 Da, respectively. Again, the relative intensities of these two signals clearly do not correspond to the original microparticle composition. This is attributed to selective degradation of the *N*-ethylcarbazole component, which produces characteristic molecular fragments at 180 and 168 Da (i.e., owing to loss of a methyl or ethyl group, respectively).

In summary, these preliminary impact ionization experiments confirm that parent cations corresponding to *N*-phenylcarbazole and either *N*-propylcarbazole or *N*-ethylcarbazole can be detected within a single microparticle. This is an important first step toward the future analysis of real PANH-based cosmic dust particles, which are likely to comprise much more complex mixtures of molecules. Moreover, both *N*-propylcarbazole and *N*-ethylcarbazole clearly exhibit a significantly greater propensity to undergo molecular fragmentation than *N*-phenylcarbazole. This suggests that quantification of the relative proportions of two or more PANH molecules within such cosmic dust based solely on the relative intensities of their corresponding parent cations is likely to be problematic under non-optimized impact ionization conditions.

## Conclusions

We report the first synthetic mimics for PANH-based cosmic dust that are sufficiently small to be suitable for impact ionization mass spectrometry studies. Two examples of hybrid PANH microparticles have been prepared: one type comprises 65 mol % *N*-ethylcarbazole and 35 mol % *N*-phenylcarbazole while the other type comprises 71 mol % *N*-propylcarbazole and 29 mol % *N*-phenylcarbazole. This corresponds to the eutectic composition in each case and ensures minimal compositional drift during processing, which is achieved via hot emulsification followed by high-pressure microfluidization. This approach produces a polydisperse spherical morphology with a mean diameter of approximately 0.80 μm—the first example of submicron-sized PANH-based hybrid microparticles with a constant (known) chemical composition. After coating with an overlayer of an electrically conductive polymer, these PANH microparticles can be accelerated up to the hypervelocity regime using a high-voltage dust accelerator. Notably, the parent cations for *N*-phenylcarbazole and either *N*-propylcarbazole or *N*-ethylcarbazole can be detected in the ionic plasma that is generated when such a microparticle strikes a gold target at 1.9 to 5.0 km s^–1^. However, the minor *N*-phenylcarbazole component is less susceptible to molecular fragmentation so its parent cation is more prominent than that of either *N*-propylcarbazole or *N*-ethylcarbazole. Such laboratory-based experiments are expected to aid the calibration of next-generation cosmic dust analyzers and hence inform the interpretation of impact ionization data anticipated from the current Europa Clipper and Interstellar Mapping and Acceleration Probe (IMAP) space missions.

## Supplementary Material



## Notes

Experimental details, including materials, synthesis and characterization methods; DSC data for a 65:35 *N*-ethylcarbazole/*N*-phenylcarbazole binary mixture; DSC data for polypyrrole-coated 65:35 *N*-ethylcarbazole/*N*-phenylcarbazole hybrid microparticles; schematic representation of the preparation of polypyrrole-coated 71:29 *N*-propylcarbazole/*N*-phenylcarbazole hybrid microparticles; optical microscopy images and laser diffraction data for 71:29 *N*-propylcarbazole/*N*-phenylcarbazole hybrid microparticles; FT-IR spectra for polypyrrole-coated 71:29 *N*-propylcarbazole/*N*-phenylcarbazole hybrid microparticles before and after acetone extraction (plus polypyrrole bulk powder spectra); impact ionization mass spectrum for a polypyrrole-coated 71:29 *N*-propylcarbazole/*N*-phenylcarbazole microparticle fired into a gold target at 1.9 km s^–1^; impact ionization mass spectrum for a polypyrrole-coated 65:35 *N*-ethylcarbazole/*N*-phenylcarbazole microparticle fired into a gold target at 5.0 km s^–1^ (PDF)
