# Supplementary material for: Synthetic Mimics for Nitrogen-Based Polycyclic Aromatic Hydrocarbon Cosmic Dust: Preparation and Preliminary Impact Ionization Mass Spectrometry Studies
Source: J Am Chem Soc. 2026 Mar 23;148(13):13592–601. doi: 10.1021/jacs.5c18079 (PMC13067273; doi:10.1021/jacs.5c18079)
Supplement: Supplementary file 1 [file ja5c18079_si_001.pdf]

Supporting Information for:

Synthetic Mimics for Nitrogen-based Polycyclic Aromatic Hydrocarbon Cosmic Dust: Preparation and Preliminary Impact Ionization Mass Spectrometry Studies

Min Zeng,[a,] Derek H. H. Chan,[a] Steven P. Armes,[a,]**

Rebecca Mikula,[b,c] John Fontanese,[b] and Zoltan Sternovsky[b,c]

[a]School of Mathematical and Physical Sciences, University of Sheffield, Brook Hill, Sheffield, South Yorkshire S3 7HF, UK.

[b]Laboratory for Atmospheric and Space Physics, University of Colorado, Boulder, Colorado 80303, USA.

[c]Smead Aerospace Engineering Sciences Department, University of Colorado, Boulder, Colorado 80303, USA.

* Author to whom correspondence should be addressed (s.p.armes@sheffield.ac.uk)

EXPERIMENTAL

Materials

N-Phenylcarbazole (98%) and *N*-propylcarbazole (95%) were purchased from Fluorochem (UK). *N*-Ethylcarbazole (97%), poly(*N*-vinylpyrrolidone) (PNVP; nominal molecular weight 360,000 g mol^{-1}), pyrrole (98%) and ammonium persulfate [$(NH_4)_2S_2O_8$, 98%] were purchased from Sigma-Aldrich (UK). Deionized water obtained from an ELGA PURELAB Option water purification system equipped with a DV25 unit was used for all experiments.

Synthesis

Preparation of 65:35 N-Ethylcarbazole/N-Phenylcarbazole Precursor Emulsion

N-Phenylcarbazole (0.60 g) and *N*-ethylcarbazole (0.90 g; 65 mol% *N*-ethylcarbazole) were weighed into a 100 mL round-bottomed flask. PNVP emulsifier (0.10 g, 0.33% w/w) and deionized water (28.40 g) were added to a separate 50 mL flask and stirred at 57 °C until the PNVP was completely dissolved. The flask containing the 65:35 *N*-ethylcarbazole/*N*-phenylcarbazole binary mixture was immersed in an oil bath set at 96 °C until both types of crystals melted to form a miscible molten liquid. At this point, the oil bath temperature was lowered to 57 °C. The aqueous PNVP solution (preheated to 57 °C) was then poured into the flask containing the molten 65:35 *N*-ethylcarbazole/*N*-phenylcarbazole binary mixture. An IKA Ultra-Turrax T-18 homogenizer equipped with a 10 mm dispersing tool was lowered down into the flask until the dispersing tool head was completely covered. High-shear homogenization of the oil/water mixture was conducted for 1 min at a stirring rate of 18,000 rpm. A volume-average droplet diameter of 62 μm was determined by laser diffraction.

Preparation of 65:35 N-Ethylcarbazole/N-Phenylcarbazole Microparticles

To prepare micron-sized hybrid PANH microparticles, the following protocol was used. The above hot 65:35 *N*-ethylcarbazole/*N*-phenylcarbazole precursor emulsion was further processed using a high-pressure LV1 microfluidizer (Microfluidics, USA). The applied pressure was 20,000 psi, and the precursor emulsion was passed once through the LV1 unit to produce micron-sized hybrid PANH microparticles. The initial temperature of the coarse emulsion was 53 °C and the final temperature of the fine emulsion was 30 °C. A volume-average diameter of 0.80 μm was determined by laser diffraction.

Preparation of Polypyrrole-coated 65:35 N-Ethylcarbazole/N-Phenylcarbazole Microparticles

The following protocol was used to coat 65:35 *N*-ethylcarbazole/*N*-phenylcarbazole hybrid PANHs microparticles of 0.80 μm diameter with a target polypyrrole overlayer thickness of ~ 8 nm. An aqueous dispersion of hybrid PANH microparticles (1.50 g) prepared using the above protocol was placed in a 100 mL glass vial and stirred at 500 rpm. Pyrrole (222.0 μL, 214.7 mg) was then added to this vial, followed by the addition of ammonium persulfate (424.0 mg; ammonium persulfate/pyrrole molar ratio = 0.58; targeted polypyrrole mass loading = 7.4 wt%). The reaction mixture was stirred for 10 min at 20 °C, and the initial milky-white suspension turned black. The resulting polypyrrole-coated 65:35 *N*-ethylcarbazole/*N*-phenylcarbazole microparticles were purified by centrifugation (2,000 rpm for 60 min at 4 °C) to remove unreacted pyrrole, spent oxidant and any PNVP-stabilized polypyrrole latex particles that might be formed in the aqueous continuous phase.[1] The sedimented microparticles were then dried in a vacuum oven at 20 °C overnight to afford a fine black powder.

Preparation of 71:29 N-Propylcarbazole/N-Phenylcarbazole Precursor Emulsion

N-Phenylcarbazole (0.48 g) and *N*-propylcarbazole (1.02 g; 71 mol% *N*-propylcarbazole) were weighed into a 100 mL round-bottomed flask. PNVP emulsifier (0.10 g, 0.33% w/w) and deionized water (28.40 g) were added to a separate 50 mL flask and stirred at 47 °C until the PNVP was completely dissolved. The flask containing the 71:29 *N*-propylcarbazole/*N*-phenylcarbazole binary mixture was immersed in an oil bath set at 96 °C until both types of crystals melted to form a miscible molten liquid. At this point, the oil bath temperature was lowered to 47 °C. The aqueous PNVP solution (preheated to 47 °C) was then poured into the flask containing the molten 71:29 *N*-propylcarbazole/*N*-phenylcarbazole binary mixture. An IKA Ultra-Turrax T-18 homogenizer equipped with a 10 mm dispersing tool was lowered down into the flask until the dispersing tool head was completely covered. High-shear homogenization of the oil/water mixture was conducted for 1 min at a stirring rate of 18,000 rpm. A volume-average droplet diameter of 67 μm was determined by laser diffraction.

Preparation of 71:29 N-Propylcarbazole/N-Phenylcarbazole Microparticles

To prepare submicron-sized hybrid PANH microparticles, the following protocol was used. The above hot 71:29 *N*-propylcarbazole/*N*-phenylcarbazole precursor emulsion was further processed using a high-pressure LV1 microfluidizer (Microfluidics, USA). The initial temperature of the coarse emulsion was 40 °C and the final temperature of the fine emulsion was 31 °C. The applied pressure was 20,000 psi, and the precursor emulsion was passed once through the LV1 unit to produce submicron-sized hybrid PANH microparticles. A volume-average droplet diameter of 0.78 μm was determined by laser diffraction.

Preparation of Polypyrrole-coated 71:29 N-Propylcarbazole/N-Phenylcarbazole Microparticles

The following protocol was used to coat 71:29 *N*-propylcarbazole/*N*-phenylcarbazole hybrid PANHs microparticles of 0.78 μm diameter with a target polypyrrole overlayer thickness of ~ 8 nm. An aqueous dispersion of hybrid PANH microparticles (1.50 g) prepared using the above protocol was placed in a 100 mL glass vial and stirred at 500 rpm. Pyrrole (222.0 μL, 214.7 mg) was then added to this vial, followed by the addition of ammonium persulfate (424.0 mg; ammonium persulfate/pyrrole molar ratio = 0.58; targeted polypyrrole mass loading = 7.4 wt%). The reaction mixture was stirred for 10 min at 20 °C, and the initial milky-white suspension turned black. The resulting polypyrrole-coated 71:29 *N*-propylcarbazole/*N*-phenylcarbazole microparticles were purified by centrifugation (2,000 rpm for 60 min at 4 °C) to remove unreacted pyrrole, spent oxidant and any PNVP-stabilized polypyrrole latex particles that might be present in the aqueous continuous phase.[1] The sedimented microparticles were then dried in a vacuum oven at 20 °C overnight to afford a fine black powder.

Characterization Methods

Melting point measurements. A series of *N*-ethylcarbazole/*N*-phenylcarbazole binary mixtures of varying composition were weighed into 7 mL glass vials and sealed with a rubber septum. Each vial was heated using a heat gun until the crystals completely melted to produce a homogenous molten liquid. Each binary mixture eventually recrystallized on standing overnight at 20 °C (some samples required stirring with a spatula to induce recrystallization). The melting points for the resulting series of *N*-ethylcarbazole/*N*-phenylcarbazole binary mixtures (*ca*. 2 mg per sample) were determined using a Stuart SMP50 automatic melting point instrument at a heating rate of 1.0 °C min^{-1}. The melting points for a series of *N*-propylcarbazole/*N*-phenylcarbazole binary mixtures were determined using the same protocol.

Laser Diffraction Particle Size Analysis. Emulsion droplets and microparticles were analyzed using a Malvern Mastersizer 3000 laser diffractometer equipped with a Hydro EV wet dispersion unit, a red He-Ne laser (λ = 633 nm) and a blue LED light source (λ = 470 nm). The stirring rate was set at 1,700 rpm and the volume-average diameter, D[4,3], was calculated from the data. Each measurement was repeated for three times.

Differential Scanning Calorimetry. Measurements were performed using a TA DSC25 Discovery series instrument operating from –35 °C to 55 °C at a heating rate of 1 °C min^{-1} using aluminum Tzero pans and Tzero hermetic lids. Calibration was performed using an indium standard. All DSC analyses involved an initial heating/cooling cycle followed by a heat ramp.

Optical Microscopy. Representative images for the two types of PANH emulsion droplets and the corresponding PANH microparticles were recorded using a Cole-Palmer optical microscope fitted with a Moticam camera linked to a PC with Motic Images Plus 3.0 software.

Scanning Electron Microscopy. Representative SEM images for the 65:35 *N*-ethylcarbazole/*N*-phenylcarbazole microparticles were recorded after coating with an ultrathin overlayer of polypyrrole using an FEI Inspect-F50 instrument at an accelerating voltage of 5 kV and a beam current of 187 µA. Samples were dried onto silicon wafers and then sputter-coated with a thin overlayer of gold to prevent sample charging.

FTIR Spectroscopy. Spectra were recorded using a Perkin-Elmer Spectrum Two FT-IR spectrometer with 16 scans being averaged per spectrum at a spectral resolution of 2 cm^{-1}.

Dust Accelerator Experiments

Impact ionization experiments were performed using the University of Colorado dust accelerator[2] following the protocols reported by Mikula *et al.*[3] An electrostatic potential of 2.2 MV was used to accelerate (sub)micron-sized microparticles. The hybrid PANH dust sample was mixed with spherical iron grains of 6-10 μm radius in a 1:1 volume ratio to reduce clumping within the source. Owing to their much lower terminal velocities, these iron particles did not reach the experimental chamber. The dust was electrostatically agitated by pulsing the source potential relative to a biased tungsten needle, which facilitated charging and extraction of the microparticles at the high-voltage terminal. Their velocity and charge were determined using two pick-up tube detectors positioned along the beamline. The particle mass was calculated by assuming a spherical morphology and helium pycnometry was used to determine the density of the PPy-coated 65:35 *N*-ethylcarbazole/*N*-phenylcarbazole microparticles (1.23 g cm^{-3}) and the PPy-coated 71:29 *N*-propylcarbazole/*N*-phenylcarbazole microparticles (1.26 g cm^{-3}). An electrostatic gate downstream allowed further selection by either mass and/or velocity.[4] Accelerator and chamber pressures were consistently below 10^{-7} and 10^{-6} Torr, respectively.

Impact ionization mass spectra were recorded using a laboratory prototype of the Interstellar Dust Experiment (IDEX) time-of-flight mass spectrometer.[5, 6] This prototype instrument has the same form factor and ion optics design as the flight unit launched on NASA's Interstellar Mapping and Acceleration Probe (IMAP) mission.[7, 8] Dust impacts occurred on a highly polished, high-purity gold-coated target biased to + 3 kV. The resulting ions were extracted and focused using a reflectron-type ion optics assembly consisting of ring electrodes and a parabolic grid. Detection was performed with a custom-made discrete-dynode electron multiplier, whose output was coupled directly to a high-bandwidth, wide dynamic range digital oscilloscope. In principle, this spectrometer has a mass resolution of $m/\Delta m \gg 200$ at 100 u.

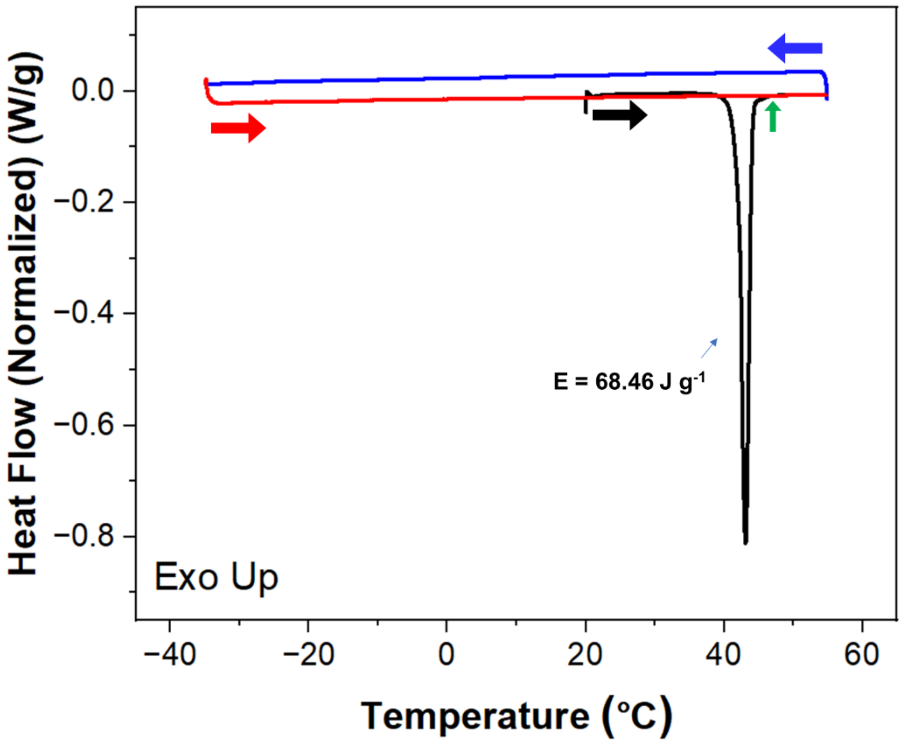

Figure S1. Differential scanning calorimetry curve recorded for a 65:35 *N*-ethylcarbazole/*N*-phenylcarbazole binary mixture (black, blue and red traces denote the initial heating run, the cooling run and the second heating run, respectively). The melting point for the 65:35 *N*-ethylcarbazole/*N*-phenylcarbazole binary mixture is 47 °C (see green arrow). Note that no recrystallization occurs during the cooling run and hence no melting transition occurs during the second heating run.

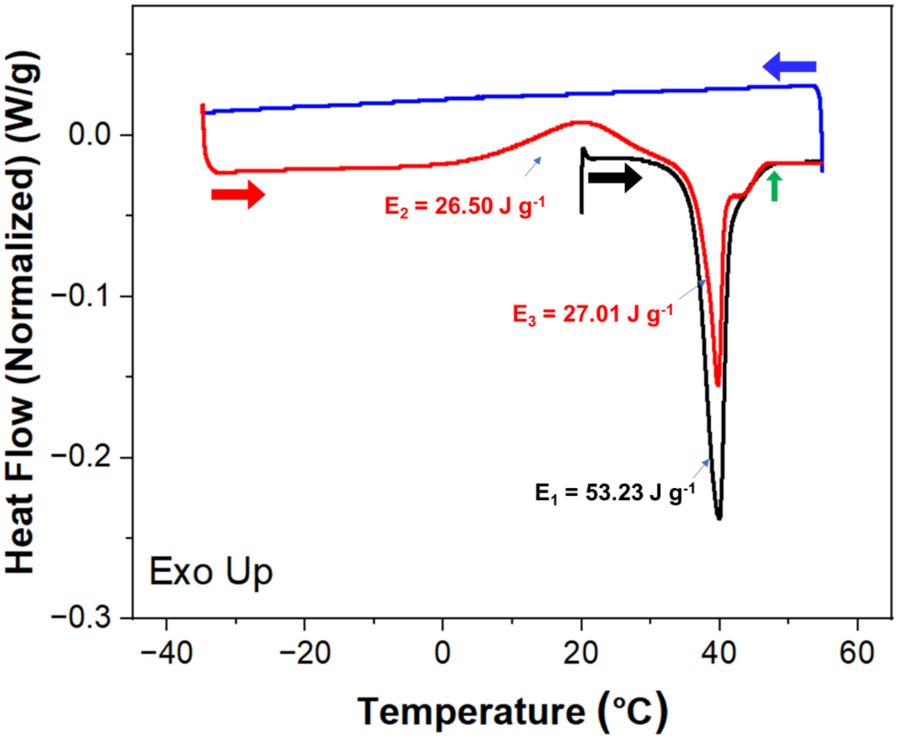

Figure S2. Differential scanning calorimetry data recorded for polypyrrole-coated 65:35 *N*-ethylcarbazole/*N*-phenylcarbazole hybrid microparticles (black, blue and red traces denote the initial heating run, the cooling run and the second heating run, respectively). The melting point for the polypyrrole-coated 65:35 *N*-ethylcarbazole/*N*-phenylcarbazole hybrid microparticles is 47 °C (see green arrow).

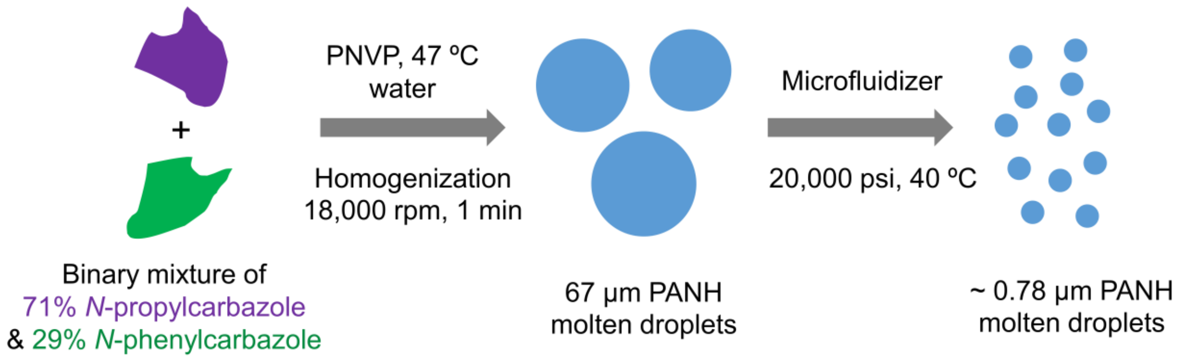

Figure S3. Schematic representation of the hot emulsification of a binary mixture comprising 71 mol% *N*-propylcarbazole and 29 mol% *N*-phenylcarbazole using poly(*N*-vinylpyrrolidone) [PNVP] as a water-soluble polymeric emulsifier at 47 °C to produce an initial coarse oil-in-water emulsion (**step 1**). Subsequent high-pressure microfluidization of this precursor emulsion at 40 °C produces a much finer emulsion comprising hybrid PANH droplets of *ca.* 0.78 μm diameter (**step 2**).

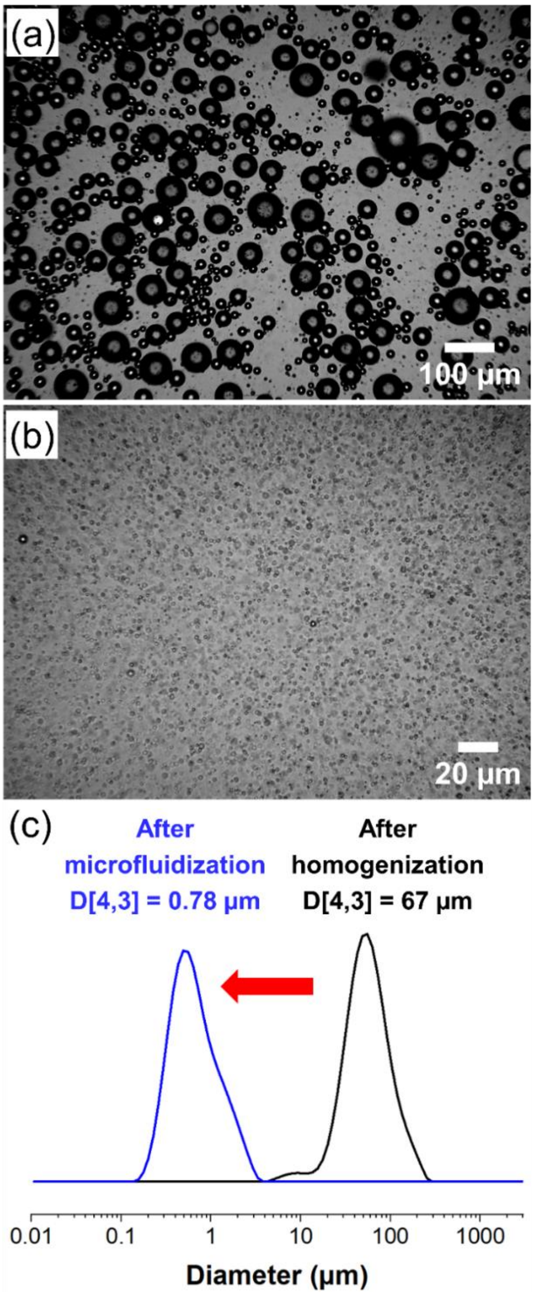

Figure S4. (a) Representative optical microscopy image recorded for the initial coarse PANH emulsion droplets (comprising 71 mol% *N*-propylcarbazole and 29 mol% *N*-phenylcarbazole) obtained after high-shear homogenization (18,000 rpm for 1 min at 47 °C). (b) Representative optical microscopy image recorded for the corresponding much finer PANH emulsion droplets obtained after high-pressure microfluidization (one pass at 20,000 psi). (c) Laser diffraction particle size distribution recorded for the initial coarse PANH droplets (black curve) and the much finer PANH droplets obtained after high-pressure microfluidization (blue curve).

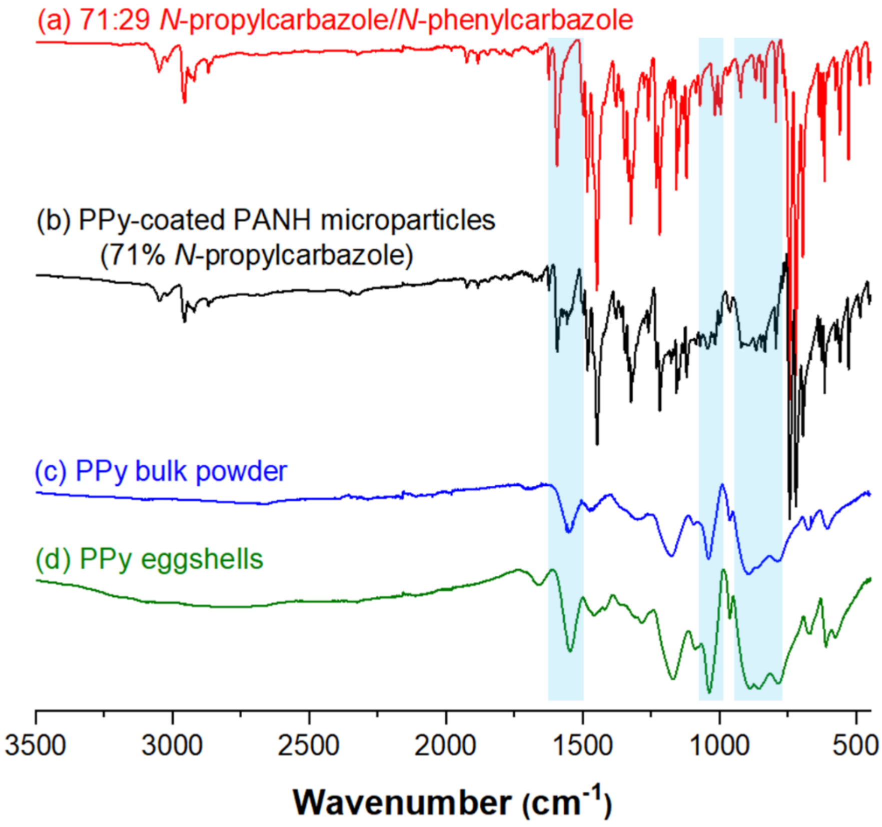

Figure S5. FT-IR spectra recorded in transmission mode for: (a) a binary mixture of *N*-propylcarbazole/*N*-phenylcarbazole crystals comprising 71 mol% *N*-propylcarbazole; (b) polypyrrole-coated 71:29 *N*-propylcarbazole/*N*-phenylcarbazole hybrid microparticles of ca. 0.78 μm diameter; (c) polypyrrole bulk powder prepared using the $(NH_4)_2S_2O_8$ oxidant; (d) residual polypyrrole eggshells obtained after selective acetone dissolution of the underlying hybrid PANH cores of the hybrid microparticles shown in (b). Such FT-IR spectra confirm that acetone extraction removed the *N*-propylcarbazole/*N*-phenylcarbazole components completely, leaving only the acetone-insoluble polypyrrole residues. A mass balance experiment conducted before and after such solvent extraction indicated a PPy mass loading of 11.9%, which corresponds to a mean PPy overlayer thicknesses of 12.4 nm.

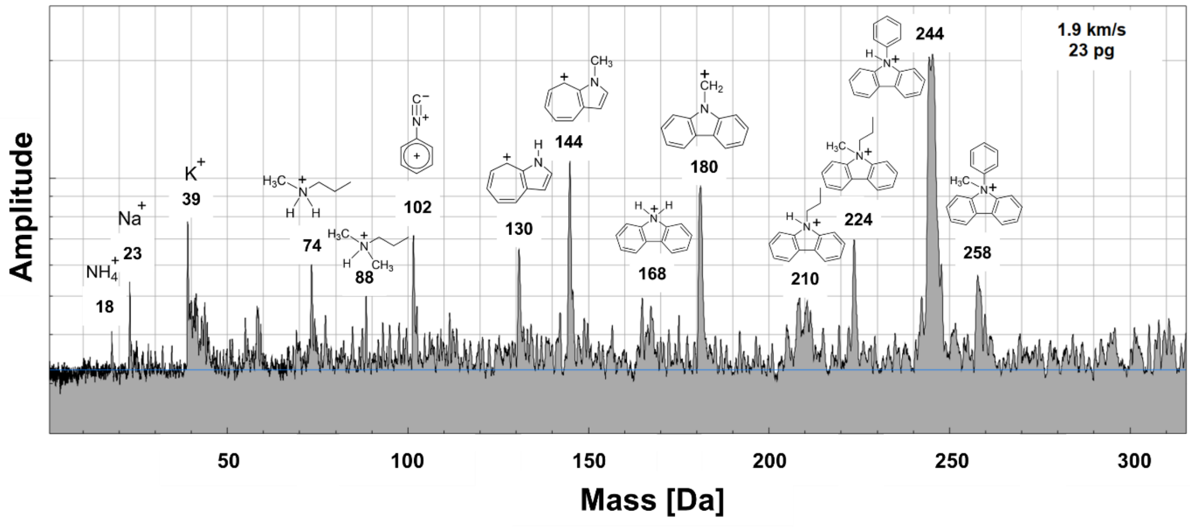

Figure S6. Impact ionization mass spectrum recorded after firing a PPy-coated 71:29 *N*-propylcarbazole/*N*-phenylcarbazole microparticle (mass = 23 picograms; mean diameter = 3.3 μm) into a gold target at 1.9 km s^{-1}. [N.B. The mass accuracy is estimated to be approximately ± 1 Da for higher mass species].

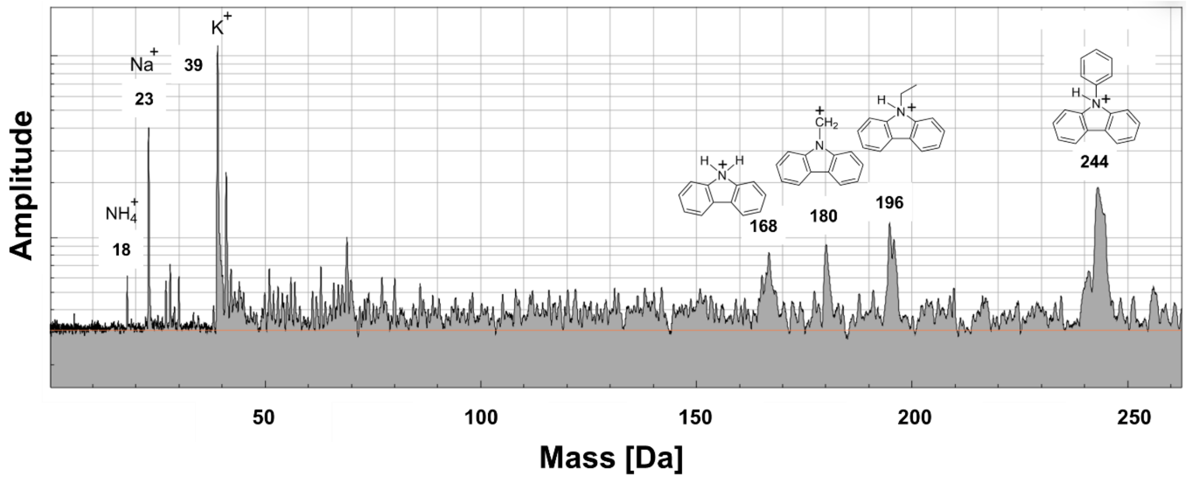

Figure S7. Impact ionization mass spectrum recorded after firing a PPy-coated 65:35 *N*-ethylcarbazole/*N*-phenylcarbazole microparticle (mass = 0.55 picograms; mean diameter = 0.95 μm) into a gold target at 5.0 km s^{-1}. [N.B. The mass accuracy is estimated to be approximately ± 1 Da for higher mass species].

REFERENCES

(1) Arms, S. P.; Vincent, B. Dispersions of electrically conducting polypyrrole particles in aqueous media. *J. Chem. Soc., Chem. Commun.* **1987**, 288-290.

(2) Shu, A.; Collette, A.; Drake, K.; Grün, E.; Horányi, M.; Kempf, S.; Mocker, A.; Munsat, T.; Northway, P.; Srama, R.; et al. 3 MV hypervelocity dust accelerator at the Colorado Center for Lunar Dust and Atmospheric Studies. *Rev. Sci. Instrum.* **2012**, *83*, 075108.

(3) Mikula, R.; Sternovsky, Z.; Armes, S. P.; Ayari, E.; Bouwman, J.; Chan, D. H. H.; Fontanese, J.; Horanyi, M.; Hillier, J. K.; Kempf, S.; et al. Impact Ionization Mass Spectra of Polypyrrole-Coated Anthracene Microparticles: A Useful Mimic for Cosmic Polycyclic Aromatic Hydrocarbon Dust. *ACS Earth Space Chem.* **2024**, *8*, 586-605.

(4) Thomas, E.; Auer, S.; Drake, K.; Horányi, M.; Munsat, T.; Shu, A. FPGA cross-correlation filters for real-time dust detection and selection. *Planet. Space Sci.* **2013**, *89*, 71-76.

(5) Sternovsky, Z.; Amyx, K.; Bano, G.; Landgraf, M.; Horanyi, M.; Knappmiller, S.; Robertson, S.; Grün, E.; Srama, R.; Auer, S. Large area mass analyzer instrument for the chemical analysis of interstellar dust particles. *Rev. Sci. Instrum.* **2007**, *78*, 014501.

(6) Sternovsky, Z.; Grün, E.; Drake, K.; Jianfeng, X.; Horanyi, M.; Srama, R.; Kempf, S.; Postberg, F.; Mocker, A.; Auer, S.; et al. Novel instrument for Dust Astronomy: Dust Telescope. In *2011 Aerospace Conference*, 5-12 March 2011, 2011; pp 1-8.

(7) McComas, D. J.; Christian, E. R.; Schwadron, N. A.; Fox, N.; Westlake, J.; Allegrini, F.; Baker, D. N.; Biesecker, D.; Bzowski, M.; Clark, G.; et al. Interstellar Mapping and Acceleration Probe (IMAP): A New NASA Mission. *Space Sci. Rev.* **2018**, *214*, 116.

(8) Horányi, M.; Tucker, S.; Sternovsky, Z.; Tyagi, K.; Knappmiller, S.; Ayari, E.; Mikula, R.; Szalay, J. R.; Kempf, S.; Bollendonk, C.; et al. Interstellar Dust Experiment (IDEX) Onboard NASA's Interstellar Mapping And Acceleration Probe (IMAP). *Space Sci. Rev.* **2025**, *221*, 102.
